# The Improved Proximal Phalanx Osteotomy for Reducing Sesamoid in Hallux Valgus Surgery—A Cadaver Study

**DOI:** 10.3390/ijerph19116487

**Published:** 2022-05-26

**Authors:** Dong-Oh Lee, Eunah Hong, Dai-Soon Kwak

**Affiliations:** 1 SNU Seoul Hospital, Seoul 03080, Korea; ronaki@naver.com; 2 Catholic Institute for Applied Anatomy, College of Medicine, The Catholic University of Korea, Seoul 06591, Korea; 2341568@naver.com

**Keywords:** hallux valgus, hallux valgus surgery, Akin proximal phalanx osteotomy, sesamoid bone

## Abstract

A metatarsal osteotomy is known to have the effect of reduction of the sesamoid. However, the reduction of the sesamoid is not always completed by a metatarsal osteotomy alone. The purpose of this cadaver study was to show that the improved technique of a modified Akin proximal phalanx osteotomy (MPO) could be helpful for the reduction of the sesamoids in hallux valgus surgery. Ten feet of cadavers were used; the cadavers had hallux valgus on both feet. The first trial of two feet underwent only the MPO. The other eight feet underwent a proximal metatarsal chevron osteotomy and MPO simultaneously. The hallux valgus angle, intermetatarsal angle, Hardy’s grade, and Smith’s grade were measured. To predict possible complications, cadavers were dissected after surgery. In the feet that underwent an MPO only, the hallux valgus angle and sesamoid position were improved. In the feet that underwent an MPO and metatarsal osteotomy, the hallux valgus deformity was completely corrected, and the sesamoid position was improved. Overall, the hallux valgus angle and intermetatarsal angle improved from 30.6 to 8.4 degrees and from 11.2 to 4.1 degrees, respectively. The sesamoid position was reduced from 5.3 to 2.5 (Hardy) and from 1.7 to 0.7 (Smith). The MPO combined with the metatarsal osteotomy were helpful for reducing the sesamoids compared to the metatarsal osteotomy only.

## 1. Introduction

A hallux valgus deformity is largely due to the instability of the first tarsometatarsal joint. As a result, the deformity consists of a medially deviated first metatarsal head with displacement of the hallux and the sesamoids. Lateral and dorsal translation of the sesamoids progress in the hallux valgus because of the tethering effect of the adductor hallucis and the transverse metatarsal ligament as well as the deforming force of the flexor hallucis longus and flexor hallucis brevis.

The recurrence of hallux valgus is one of the common postoperative complications after corrective surgery and is of immense concern [1,2]. Residual subluxation of the sesamoids is known as a risk factor for the recurrence of hallux valgus [3,4]. Moreover, recent studies suggested that the residual abnormal position of the sesamoids affect even functional outcomes and patients’ satisfaction after hallux valgus surgery [5]. Thus, the previous studies emphasized that proper reduction of the sesamoids is important in hallux valgus surgery [6,7,8].

However, reducing the sesamoid is not always completed with a metatarsal osteotomy alone although a metatarsal osteotomy is known to have the effect of reduction of the sesamoid in and of itself. Nevertheless, there were a few methods, to the best of our knowledge, to address the residual subluxated sesamoids even after metatarsal osteotomy. Thus, the purpose of this study was to simulate the improved technique of the modified Akin proximal phalanx osteotomy (MPO) and to check the effect of this procedure on the reduction of the subluxated sesamoid. Henceforth, it is hypothesized that the MPO could help to reduce the sesamoid in hallux valgus surgery.

## 2. Materials and Methods

This study included 5 fresh cadavers (10 feet), which had hallux valgus on both the feet and no history of previous surgery on them. The mean age was 84 years (70–94 years); 6 were female, and 4 were male. The authors cut the cadavers above the supramalleolar level. This cadaveric study was exempted from obtaining consent from the institutional review board because it did not involve human subjects.

We tried the MPO only without metatarsal osteotomy to 1 cadaver (2 feet) to simulate the genuine effect of modified proximal phalanx osteotomy on the reduction of the sesamoid. However, we could not completely address the sesamoid subluxation with the MPO only because the medially deviated metatarsal head blocked the reduction of the sesamoid. Therefore, the other 4 cadavers (8 feet) underwent a proximal metatarsal chevron osteotomy and MPO simultaneously. Before and after surgery, radiographic evaluations of all cadavers were performed.

To predict complications after surgery, some maneuvers were performed. First, manual twitching was applied by one examiner, who was an orthopaedic surgeon with several years of experience, to the proximal phalanx after fixation of the osteotomy to check the stability of the osteotomy site, and the stability was compared with that of the metatarsal osteotomy site. Second, at the end of the study, all specimens were dissected, and we checked whether there was any possible injury to other normal structures.

**Figure 1:**
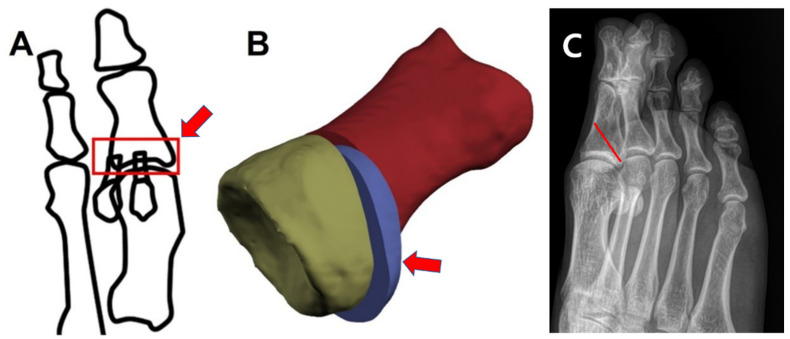
Schematic drawing of the modified proximal phalanx osteotomy. (**A**) The red rectangle shows the insertion of the flexor hallucis brevis, which is rotated for the reduction of sesamoids in an MPO; (**B**) Closed wedge osteotomy can be done; (**C**) Oblique cutting line can be visualized in the foot oblique radiograph.

The theoretical basis of the MPO is that supination of the insertion of the flexor hallucis brevis can be helpful for further rotation of the sesamoid, which the flexor hallucis brevis bears (Figure 1).

### 2.1. Surgical Technique

**Figure 2:**
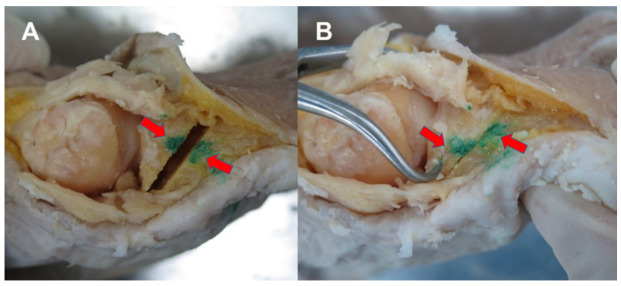
Modified proximal phalanx osteotomy performed by (**A**) an oblique medial closed wedge osteotomy for correcting hallux valgus; (**B**) rotation of the distal segment for reducing the sesamoid bones.

A single medial longitudinal incision of about 10 cm in length was made along the medial surface of the first ray from the first tarsometatarsal joint to the interphalangeal joint of the hallux, and the technique was adapted from the report of Jung et al. [9]. The medial capsule was incised, and the lateral soft tissue was released. A proximal metatarsal chevron osteotomy and fixation with two 1.6 mm Kirschner wires were performed. Subsequently, an MPO was performed, and the first cut plane went from the distal dorsum of the midshaft of the proximal phalanx to the proximal plantar cortex, 2–3 mm ahead of the proximal plantar articular margin of the proximal phalanx (Figure 1). The second cut’s plane was parallel to and 2–3 mm ahead of the first osteotomy line because this procedure was a closed wedge osteotomy on a dorsoplantar view, similar to the Akin osteotomy. By removing the segmental wedge bone, the gap of the osteotomy site was closed (Figure 2A). Then, a proximal segment of the proximal phalanx was held by a reduction clamp, and the distal portion of the hallux was supinated with a hand (Figure 2B). After the fixation of the osteotomy site with two 1.2 mm Kirschner wires, a medial capsular plication was performed at the end of the procedure.

### 2.2. Radiographic Evaluation

Before the surgery, one author held both the feet of the cadaver during radiographic imaging to mimic the standing position. A similar process was performed by the same author after the surgery.

To determine the effect of the hallux valgus correction, hallux valgus angles (HVA) and 1st−2nd intermetatarsal angles (IMA) were measured at the preoperative and postoperative period. With regard to the sesamoid position, 2 classification systems were used: (1) Hardy and Clapham’s tibial sesamoid 7 position system in a dorsoplantar view [10,11] and (2) the tibial sesamoid 4 position grade in a radiographic tangential view described by Smith et al. [11,12].

The authors postulated that the sesamoid position is within an acceptable range when the sesamoid is reduced to a position less than a 3 grade based on the classification by Hardy and Clapham [3,10].

## 3. Results

**Figure 3:**
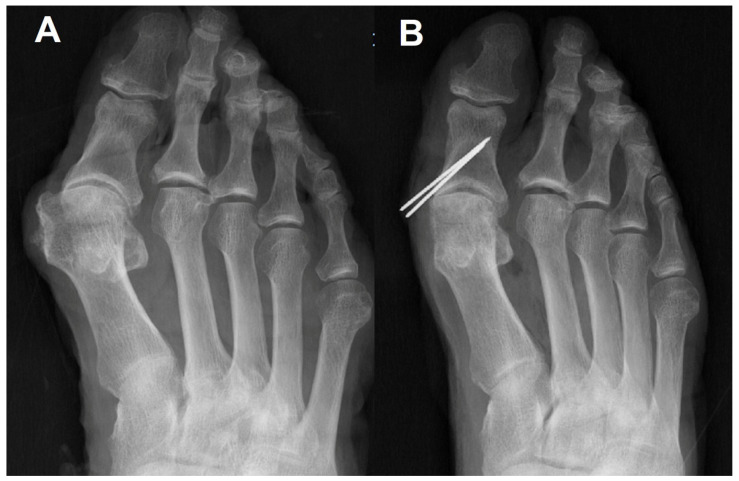
Proximal phalanx osteotomy alone reduces sesamoids to some degree but not perfectly. (**A**) Preoperative simple radiograph of hallux valgus in cadaver; (**B**) Modified proximal phalanx osteotomy was performed. Bunionectomy was done for making K-wire entry point.

The preoperative/postoperative HVA and IMA are shown in Table 1. Sesamoid positions before/after surgery are demonstrated in Table 2. All radiographic measurements in all the specimens were improved after surgery (Figure 3).

In intraoperative fluoroscopy, the sesamoid was not reduced even after the metatarsal osteotomy and medial capsular imbrications. Under this situation, a reduction of the sesamoid after manually pronating the hallux predicted that performing the MPO instead of the Akin osteotomy could be helpful in reducing the sesamoid. Finally, reduced sesamoids were observed after the MPO was performed on a standing simple radiograph as we predicted in the operating room.

The proximal phalanx osteotomy site was stable with manual stress, and the stability was comparable with that of the proximal metatarsal osteotomy site. Meticulous dissection of a cadaver at the end of the study confirmed no damage of the articular surface of a proximal segment of the proximal phalanx. However, the osteotomy plane of the first case (1R), one specimen that only underwent the MPO, was too distal to separate the insertion of the flexor hallucis brevis from the proximal fragment (Table 2). As a result, the sesamoid of this specimen did not show a considerable change in position as compared with the others (Table 2).

## 4. Discussion

Lateral translation of the sesamoids indicates a presence of deforming forces of the flexor hallucis longus and brevis as well as the tether by the transverse metatarsal ligament. Thus, residual subluxation of the sesamoids can be a risk factor for the recurrence of hallux valgus after corrective surgery. A complete reduction of the sesamoids is desirable; however, there are few procedures that reduce the residual subluxation of the sesamoids even after a corrective metatarsal osteotomy.

This study showed a reduction of the sesamoids with a proximal metatarsal osteotomy and MPO. The proximal phalangeal osteotomy fashioned with an oblique cut for the correction of hallux valgus was first introduced in 2003 [13]. However, available proximal phalangeal osteotomies were for correcting residual hallux valgus or hallux valgus interphalangeus. On the other hand, the MPO in the current study can be employed for correcting not only hallux valgus but also pronated sesamoids.

Previous procedures for reducing the sesamoids dealt with soft tissue management, especially capsular handling. A study stated that dorsolateral capsulotomy should be performed in cases where subluxation of the sesamoids remains after metatarsal osteotomy. Theses soft tissue procedures only release the lateral side of the sesamoids and imbricate the medial side of the sesamoids; they are not powerful correctional procedures for the subluxated sesamoids. However, the authors believe that the MPO is helpful for reducing the sesamoid as compared to soft tissue procedures because the MPO directly supinate the sesamoid by rotating the distal fragment, including the insertion site of the flexor hallucis brevis embedding the sesamoid.

It is known that metatarsal osteotomy, both distal and proximal osteotomy, can help to bring the sesamoids under the first metatarsal head. However, there are differences in correction rates across the studies, and sometimes, the complete reduction of the sesamoids is a failure [14,15,16,17]. The Akin osteotomy is not helpful for the reduction of the sesamoids even though it has been widely used to address residual hallux valgus deformity when it remains even after corrective metatarsal osteotomy. Instead of an Akin osteotomy, an MPO may be helpful under this kind of situation but must be validated by further research.

One of the concerns in performing the MPO is the question of whether an osteotomy harms any other normal structures we do not want to damage (the proximal articular surface) or successfully separates the insertion of the flexor hallucis brevis from the proximal segment. In our series, there was no specimen with damage on the proximal articular surface, and there was one specimen that failed to separate the insertion of the flexor hallucis brevis from the proximal segment because the osteotomy was too distal from the proximal articular surface.

There are several limitations in our research work. First, we could not use computed tomography or weight bearing radiographs to evaluate the position of the sesamoids and, instead, mainly used a dorsoplantar view of the Hardy and Clapham. Grading based on a tangential view is known to be unreliable because it depends on the crista, which may be indistinguishable due to excessive erosion, and different degrees of dorsiflexion of the metatarsophalangeal joint may modulate the sesamoid position [11,18]. Second, the MPO is not yet validated for the stability because manual testing is not objective and has no reliability. Third, this study only used cadavers without a clinical series. Moreover, evaluation of the influence of an MPO on sesamoid reduction would have been better evaluated comparing cases of metatarsal osteotomy associated with and without the MPO. However, we could not address the sesamoid subluxation with only the MPO in the first two cases at the beginning of the study because the medially deviated metatarsal head blocked the reduction of the sesamoid. Thus, we combined metatarsal osteotomy with MPO in the following eight cases. Nevertheless, we believe this outcome is meaningful because our cases show a good reduction of the sesamoid when we consider that metatarsal osteotomies alone are not always perfect in reducing the sesamoid.

## 5. Conclusions

The MPO in the correction surgery of hallux valgus may be clinically helpful for reducing the sesamoids because the MPO combined with metatarsal osteotomy reduced the sesamoids well compared to metatarsal osteotomy alone in this cadaver study. Future studies would be needed to collect numerous clinical cases that have the residual subluxation of the sesamoids after corrective metatarsal osteotomy.

## Figures and Tables

**Table 1:** Hallux valgus angles (HVA) and 1st−2nd intermetatarsal angles (IMA) before/after the Akin osteotomy and proximal metatarsal osteotomy, except for specimen 2 (unit: degrees).

Specimen	Preop HVA	Postop HVA	Preop IMA	Postop IMA
1R *	40.0	25.1	11.8	9.8
1L *	40.0	18.9	13.4	11.7
2R	21.8	4.0	12.7	1.7
2L	25.2	1.6	12.2	1.7
3R	29.4	6.5	9.5	6.5
3L	25.4	6.1	11.6	2.0
4R	28.7	2.1	8.8	2.0
4L	28.4	4.1	8.1	0.0
5R	40.1	10.0	13.6	4.0
5L	26.7	6.0	9.8	2.0
Mean	30.6	8.4	11.2	4.1

* Only the improved technique of the proximal phalangeal osteotomy was performed on 2 specimens.

**Table 2:** Position changes of the sesamoid before/after the MPO and proximal metatarsal osteotomy (except for specimen 2), based on 2 classifications: Hardy and Clapham’s tibial sesamoid 7 position system (H) and Smith’s tangential 4 position (S). The sesamoid position of the 1R specimen did not improve because the proximal phalanx was cut distally.

Specimen	Preop H	Postop H	Preop S	Postop S
1R *	5	3	1	1
1L *	6	5	2	1
2R	6	2	1	0
2L	5	2	1	0
3R	5	2	1	1
3L	5	3	1	1
4R	7	2	3	0
4L	5	3	3	1
5R	5	2	3	1
5L	4	1	1	1
Mean	5.3	2.5	1.7	0.7

* Only the improved technique of the proximal phalangeal osteotomy was performed on 2 specimens.

## Data Availability

The data presented in this study are available on request from the corresponding author.
